# Utility of BMIQ, a novel web‐based weight management programme, at an academic weight management centre

**DOI:** 10.1002/osp4.393

**Published:** 2019-12-04

**Authors:** Sarah R. Barenbaum, Sherin E. Mathews, Katherine H. Saunders, Guadalupe Minero, Elizabeth Mauer, Louis J. Aronne, Alpana P. Shukla

**Affiliations:** ^1^ Comprehensive Weight Control Center, Division of Endocrinology Diabetes, and Metabolism, Weill Cornell Medicine New York New York; ^2^ Department of Healthcare Policy and Research Weill Cornell Medicine New York New York; ^3^ BMIQ Greenwich Connecticut

**Keywords:** online behavioural programme, anti‐obesity pharmacotherapy, weight loss, BMIQ

## Abstract

**Background:**

BMIQ is a customizable online platform used to deliver evidence‐based behavioural management that complements office visits with healthcare providers. BMIQ offers educational materials, meal plans and tracking tools for patients, and remote monitoring and guidance for physicians. In this retrospective chart review, the implementation and utility of BMIQ was assessed in patients treated at the Comprehensive Weight Control Center (CWCC) at Weill Cornell Medicine.

**Methods:**

The study included all new patients seen at the CWCC between 9/1/2016 and 6/1/2017 who enrolled in BMIQ. Use of BMIQ was assessed by the number of enrolled patients who activated their account and viewed BMIQ sessions. Demographics, comorbidities, medications, and weight change during 6‐month follow‐up were obtained from the electronic medical records.

**Results:**

Of the 495 new patients enrolled in BMIQ, 217 met the inclusion criteria of 6‐month follow‐up. The mean age was 50.2 (±13.1) and 72% (n = 157) were female. Sixty‐four percent (n = 138) activated their BMIQ account and viewed greater than or equal to 1 BMIQ session. The average number of physician and registered dietitian visits were 3.5 (±1.1) and 1.9 (±1.6), respectively. The average number of weight loss medications was 1.6 with metformin being the most commonly prescribed (76%). Mean weight loss at 6 months was 7.0 ± 5.9%; 59% achieved greater than or equal to 5% weight loss and 28% achieved greater than or equal to 10% weight loss. The total number of physician visits and weight‐loss pharmacotherapies used were significant predictors of patients achieving greater than or equal to 5% weight loss. Regular BMIQ usage was an independent predictor of patients achieving both greater than or equal to 5% and greater than or equal to 10% weight loss at 6 months.

**Conclusion:**

Clinically significant weight loss was achieved in the majority of patients with limited in‐clinic physician and registered dietitian follow‐up in combination with BMIQ. This retrospective study demonstrates the utility of online behavioural therapy as part of a medical weight management intervention.

## 1 BACKGROUND

Obesity has become an alarming epidemic in the United States. With over 71% of American adults over the age of 20 suffering from overweight or obesity, there is an urgent need for practical, comprehensive interventions with broad reach.^1^ Patients with overweight and obesity are at increased risk of multiple morbidities including cardiovascular disease, stroke, type 2 diabetes, osteoarthritis, obstructive sleep apnea, and all‐cause mortality.^2^ Meanwhile, weight loss of 5% to 10% among these patients is sufficient for clinically significant improvements in health outcomes.^3^, ^4^

Lifestyle interventions including diet, exercise, and behavioural modification remain the cornerstone of weight management. The intensive lifestyle interventions delivered in the Action for Health in Diabetes (Look AHEAD) and Diabetes Prevention Program (DPP) studies are examples of successful programmes that provide face‐to‐face counseling on behavioural modification.^5^, ^6^ The Practice‐based Opportunities for Weight Reduction (POWER) trials, which consisted of three independent but coordinated randomized controlled trials, were among the first to study the benefits of intensive behavioural interventions in the primary care setting.^7^, ^8^, ^9^ While the studies delivered a range of interventions either in‐person or remotely, the common finding was that the most successful interventions had more frequent patient follow‐up, regardless of delivery method. These studies demonstrated the success of intensive behavioural interventions and suggested the utility of remote delivery.^10^ A more recent randomized controlled trial illustrated that intensive behavioural therapy (IBT) delivered by primary care physicians over 21 brief sessions led to clinically meaningful weight loss at 1 year.^11^ The addition of liraglutide 3.0 mg to IBT nearly doubled weight loss in this study. Due to practical, economic, and logistical reasons, however, in‐person IBT is challenging to implement for many of the patients who would benefit from it, ultimately making it unlikely to succeed as a broad intervention for obesity in this country.

Ninety percent of American adults have access to the internet, making online weight management programmes attractive as convenient, cost effective, and scalable tools to combat the obesity epidemic.^12^, ^13^, ^14^ Online weight management programmes hold particular promise for target populations that have traditionally been considered difficult to reach (eg, due to rural locations, lower income, or inability to meet the time and travel constraints of IBT).^15^, ^16^ Interactive computer‐based interventions are more effective than no treatment; however, they have not been shown to be as effective as face‐to‐face counseling.^17^ In practice, these tools may be most effective as part of a broader weight loss programme, used in conjunction with clinician guidance.^17^ Despite the growing body of literature supporting the utility of online weight management programmes, there is little data related to the utility of such programmes delivering lifestyle interventions when used specifically in combination with limited in‐person treatment and pharmacotherapy at tertiary weight management centres.

This study was a retrospective chart review assessing the adoption and utility of BMIQ, a novel web‐based weight management programme offered to patients at the Comprehensive Weight Control Center (CWCC) at Weill Cornell Medicine in conjunction with pharmacotherapy and in‐person treatment. The CWCC is an academic tertiary weight management centre comprising physicians (MDs), nurse practitioners (NPs), and registered dietitians (RDs). Most patients met with an RD at least once, and the majority of patients were prescribed antiobesity pharmacotherapy. The study hypothesis was that regular use of BMIQ (four or more sessions) in combination with in‐person treatment would result in greater weight loss than in‐person treatment with minimal use of BMIQ (<4 sessions).

## 2 METHODS

BMIQ is a customizable online platform designed for two distinct audiences: patients and providers. For patients, BMIQ delivers evidence‐based behavioural management that complements office visits with healthcare providers.^18^ Patients can view over 30 tutorial videos (referenced as sessions) on topics surrounding diet, physical activity, and lifestyle modification. BMIQ also provides patients with comprehensive meal plans and a variety of detailed reading material that further expands on the topics covered in each session. Patients have access to integrated weight and goal‐tracking tools, which providers can also monitor remotely. Providers also receive guidance on managing patients via customized plans based on the output from a patient‐completed behaviour and health assessment. To facilitate collaboration between patients and providers, the platform also offers messaging functionality.

All new patients seen in person at the CWCC between 9/1/2016 and 6/1/2017 were identified by an Epic query, and the electronic medical records of those patients who were enrolled in BMIQ were reviewed. The following data were queried from BMIQ: total number of enrolled patients who activated their account, login rates, and completion of BMIQ sessions. Demographic data and medical history were recorded as well as antiobesity medications, weight, and body mass index (BMI) at baseline and at 6‐month follow‐up. Exclusion criteria included loss to follow‐up at 6 months and patients who only saw an RD and not a physician.

Descriptive statistics were used to characterize the secondary outcomes of weight and BMI using mean and standard deviation; paired *t* tests and Wilcoxon signed‐rank tests were used to assess changes from baseline to 6 months. Weight loss outcomes were compared between patients who met the threshold of completing four or more online BMIQ sessions and those who did not meet this threshold. Multivariate modelling was implemented to evaluate the independent impact of BMIQ use on patients achieving greater than or equal to 5% and greater than or equal to 10% weight loss after controlling for demographic and clinical variables of interest (including age, gender, comorbidities, and use of antiobesity pharmacotherapy). *P* values were two‐sided with statistical significance evaluated at the .05 alpha level.

The study was approved by the Weill Cornell Medical College Institutional Review Board (IRB protocol #1710018679R001).

## 3 RESULTS

Four hundred and ninety‐five new patients seen by physicians at the CWCC were enrolled in BMIQ during the study period, of whom 217 had 6‐month follow‐up. Sixty‐four percent of these new patients (n = 138) activated their BMIQ account and viewed greater than or equal to 1 BMIQ session. Forty‐eight percent (n = 66) of patients who activated BMIQ viewed greater than or equal to four BMIQ sessions. In the cohort who viewed greater than or equal to four BMIQ sessions, the average number of sessions viewed was 10.8 ± 5.7. In the cohort who viewed less than four BMIQ sessions, the average number of sessions viewed was 0.78 ± 0.8. Mean age was 50.2 **±** 13.1 years, 72% of the patients were female, 12% of all patients had type 2 diabetes, and 52% had prediabetes. Mean weight was 103.4 **±** 24.1 kg, and mean BMI was 36.4 **±** 6.9 kg/m^2^. The average number of physician and RD visits was 3.5 ± 1.1 and 1.9 ± 1.6, respectively.

The majority of patients were prescribed antiobesity pharmacotherapy including FDA‐approved and off‐label medications. At 6 months, the patients were prescribed an average of 1.6 medications for weight loss; the most commonly prescribed were metformin (76%), bupropion (26%), liraglutide (17%), and naltrexone (17%).

Mean weight loss at 6 months was 7.0 **±** 5.9% (*P* < .001 compared with baseline); 59% achieved greater than or equal to 5% weight loss and 28% achieved greater than or equal to 10% weight loss (Table 1). Patients who viewed greater than or equal to four BMIQ sessions averaged greater weight loss than those viewed fewer than 4 sessions (8.7 ± 6.6% vs 6.3 **±** 5.5%; *P* = .011). For those who viewed greater than or equal to four BMIQ sessions, 70% achieved greater than or equal to 5% weight loss and 41% achieved greater than or equal to 10% weight loss, compared to patients who viewed fewer than four sessions of whom 54% achieved greater than or equal to 5% weight loss and 22% achieved greater than or equal to 10% weight loss (*P* = .027 at 5%; *P* = .0038 at 10%). Of the cohort who viewed greater than or equal to four BMIQ sessions, those who saw the RD (in addition to the physician) had similar outcomes to those who did not see the RD (Table 1).

**Table 1:** Weight loss outcomes by cohort

	n	Mean Weight Loss % (SD)	% Achieving ***≥***5% Weight Loss	% Achieving ***≥***10% Weight Loss
All patients with 6‐month physician follow up	217	7.0 (5.9)*	59	28
<4 BMIQ sessions	151	6.3 (5.5)* ^,^ **	54**	22*
4+ BMIQ sessions	66	8.7 (6.6)* ^,^ *	70*	41*
4+ BMIQ sessions + ≥1 registered dietician visit	47	9.8 (6.8)*	77	49

Abbreviation: SD, standard deviation.

* *P* < .05 vs baseline.

** *P* < 0.05 for <4 BMIQ sessions vs 4+ BMIQ sessions.

Finally, in multivariate modelling, the total number of physician visits and weight‐loss pharmacotherapies used were significant predictors of patients achieving greater than or equal to 5% weight loss. Regular BMIQ usage was an independent predictor of patients achieving both greater than or equal to 5% (*P* = .042) and greater than or equal to 10% (*P* = .006) weight loss at 6 months (Table 2).

**Table 2:** Logistic regression by predictor variable

	≥5% Weight Loss				≥10% Weight Loss			
			95% CI				95% CI	
Predictor variable	*P* Value	OR	Lower	Upper	*P* Value	OR	Lower	Upper
Age	.053	1.023	1.000	1.047	.054	1.026	1.000	1.052
Male gender	.098	1.783	0.900	3.532	.133	1.705	0.850	3.419
Initial BMI	.607	0.988	0.943	1.035	.328	1.025	0.976	1.077
MD visits	.011	1.463	1.091	1.963	.103	1.282	0.951	1.729
RD visits	.427	1.075	0.899	1.287	.130	1.156	0.958	1.396
≥4 BMIQ sessions	.013	2.315	1.193	4.493	.006	2.547	1.309	4.958
Number of AOMs	.042	1.390	1.012	1.910	.922	1.017	0.723	1.432

Abbreviations: AOM, antiobesity medication; BMI, body mass index; CI, confidence interval; OR, odds ratio.

## 4 DISCUSSION

The principal finding of this study was that online behavioural management complemented office visits with healthcare providers and promoted greater weight loss. In this retrospective review, patients who viewed at least four BMIQ sessions achieved superior weight‐loss outcomes compared with those who did not. More frequent encounters with a physician predicted greater weight loss. This was consistent with previously published literature, which has shown that greater patient‐provider contact predicted better weight loss outcomes.^10^, ^11^, ^19^ However, in contrast to the existing studies, there were significantly fewer face‐to face visits with a provider in this study.

The use of multiple pharmacotherapies also predicted greater weight loss. Combination therapies have been shown to have additive effects by targeting different signalling pathways involved in the regulation of body weight.^20^, ^21^ Of note, in this study, the most commonly prescribed medication was metformin, which was partly driven by the high rate of prediabetes in this cohort. Although not FDA‐approved for obesity, data from both experimental and clinical studies provide sound rationale for its use for weight management.^22^, ^23^ The average number of weight loss pharmacotherapies used across the study groups was similar; however, weight loss was greater in those patients who viewed four or more BMIQ sessions. This finding suggested a benefit of online behavioural support beyond pharmacotherapy but needs to be confirmed in prospective studies.

Ultimately, this study illustrated that clinically significant weight loss of 9% to 10% can be achieved with a limited number of physician and RD visits when combined with regular online behavioural management support and pharmacotherapy. This model at the CWCC combined elements that have individually been shown to have success in patients in a novel way that can have a significant and broad impact on the obesity epidemic. Given the relatively low in‐office time involved, this may be a practical approach to target difficult‐to‐reach populations.

This study had several limitations, including the retrospective nature of the design, short period of follow‐up, and small sample size of patients. Although a positive signal was observed on multivariable modelling, it is not possible to clearly isolate the benefit of the programme beyond pharmacotherapy due to the lack of a control group. This warrants further study with longer follow‐up in a larger prospective cohort to clearly delineate the effectiveness of this programme.

## 5 CONCLUSION

Regular use of BMIQ in addition to limited office visits with a physician resulted in improved weight loss outcomes. This retrospective study demonstrated the utility of online behavioural therapy as part of a medical weight management intervention.

### CONFLICT OF INTERESTS

Katherine H. Saunders has an ownership and management interest in BMIQ. Guadalupe Minero is an employee of and has an ownership interest in BMIQ. Louis J. Aronne is the founder and CEO of BMIQ. All other authors have declared no conflicts of interest.

## AUTHOR CONTRIBUTIONS

Study concept and design: A.P.S. and S.R.B.

Data acquisition: S.R.B., S.M., G.M., and K.S.

Data analysis: E.M., S.R.B., S.M., and A.P.S.

Data interpretation: A.P.S., S.R.B., and L.J.A.

Drafting of manuscript: S.R.B. and A.P.S.

Review and edit manuscript: all authors.
